# Visual Detection of Triethylamine and a Dual Input/Output Logic Gate Based on a Eu^3+^-Complex

**DOI:** 10.3390/molecules26113244

**Published:** 2021-05-28

**Authors:** Bao-Ning Li, Yuan-Yuan Liu, Ya-Ping Wang, Mei Pan

**Affiliations:** MOE Laboratory of Bioinorganic and Synthetic Chemistry, Lehn Institute of Functional Materials, School of Chemistry, Sun Yat-sen University, 135 West Xingang Road, Guangzhou 510275, China; libn7@mail2.sysu.edu.cn (B.-N.L.); liuyy253@mail2.sysu.edu.cn (Y.-Y.L.); yapingwang0393@163.com (Y.-P.W.)

**Keywords:** Eu^3+^-complex, TEA detection, logic gate

## Abstract

A series of Ln^3+^-metal centered complexes, Ln(TTA)_3_(DPPI) (Ln = La, **1**; Ln = Eu, **2**; Ln = Tb, **3**; or Ln = Gd, **4**) [(DPPI = *N*-(4-(1H-imidazo [4,5-f][1,10]phenanthrolin-2-yl)phenyl)-*N*-phenylbenzenamine) and (TTA = 2-Thenoyltrifluoroacetone)] have been synthesized and characterized. Among which, the Eu^3+^-complex shows efficient purity red luminescence in dimethylsulfoxide (DMSO) solution, with a Commission International De L’ Eclairage (CIE) coordinate at x = 0.638, y = 0.323 and *Φ*_Eu_^L^ = 38.9%. Interestingly, increasing the amounts of triethylamine (TEA) in the solution regulates the energy transfer between the ligand and the Eu^3+^-metal center, which further leads to the luminescence color changing from red to white, and then bluish-green depending on the different excitation wavelengths. Based on this, we have designed the IMPLICATION logic gate for TEA recognition by applying the amounts of TEA and the excitation wavelengths as the dual input signal, which makes this Eu^3+^-complex a promising candidate for TEA-sensing optical sensors.

## 1. Introduction

Stimulus-response materials, which are new types of intelligent materials, have received widespread attention [1,2,3,4]. Molecular systems capable of exhibiting color-tunable luminescence in suitable substrates or under specific acid or base conditions are highly appreciated, since they favor sensors [5,6], molecular logic gates and fluorescence ratio meters, etc. [7]. Among which, lanthanide-based complexes are of particular interest owing to their specific luminescent properties, which can be applied in various areas, such as pure red organic light-emitting diodes [8], cellular recognition [5], fluorescence sensors [9,10,11], magnetic materials and catalysis [12]. As to the potential applications of stimulus-responsive luminescent complexes [13,14,15], there has been an unremitting pursuit of new organo-Eu^3+^ complexes to achieve desirable fluorescence responses for micro-environment detection [16,17,18]. Owing to the unique f-f radiative transitions, lanthanide based emitters show sharp and narrow emission bands, which usually have stability towards the surrounding environments [19]. In suitable cases, by regulating the energy of the ligand and the outer stimulations, the energy transfer between the ligands and the Ln-metal centers can be controlled, leading the emission to be “Off-On”, or leading to a change in color and intensity [20], which leads to the emission becoming a favorable and promising candidate for visual detection [21,22]. Furthermore, the detection of toxic triethylamine (TEA) [23] possesses a direct relevance to the quality of the environment and our lives and is highly demanding [24]. However, the development of stimuli-responsive systems that show a wide range of luminescent color changes for sensitive and eye-catching observations upon TEA stimulations are still worth studying [17,25,26].

**Scheme 1:**
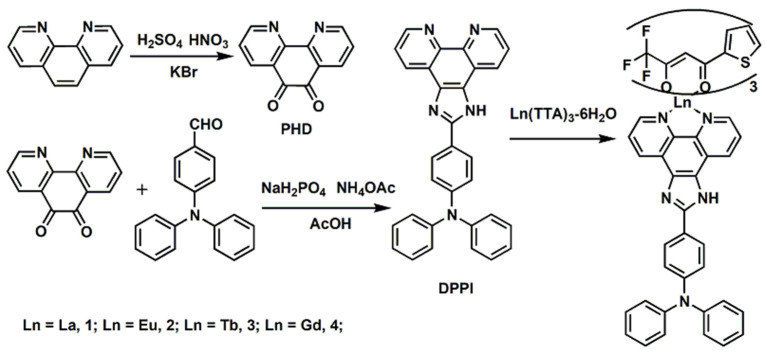
Reaction procedure for the synthesis of DPPI, and complexes **1**–**4**.

Herein, we developed a series of organo-Ln^3+^ (Ln = La, Eu, Tb, Gd) complexes coordinated with the donor-acceptor structured DPPI ligand (Scheme 1), which possesses an ILCT (intra-ligand charge transfer) potential due to the combination of phenanthroline, imidazole and triphenylamine groups, which can serve as sensitizers for the Eu^3+^-centered emission and also stimulation sites for the detection of TEA. As a result, the molecular Eu^3+^-complex showed good photophysical responses to both TEA concentrations and excitation wavelengths, and was applied well within the dual input/output logic gate design.

## 2. Results and Discussion

### 2.1. Synthesis and Photophysical Properties of the Ln-Complexes

The ligand DPPI was synthesized according to the typical reaction [27], as shown in Scheme 1. Further into its self-assembly with a deprotonated TTA ligand and LnCl_3_⋅6H_2_O (Ln = La, Eu, Tb, or Gd), a series of Ln (TTA)_3_(DPPI) complexes (Ln = La, **1**; Ln = Eu, **2**; Ln = Tb, **3**; or Ln = Gd, **4**) were obtained and well-characterized by EA, FT-IR, ^1^H NMR and ESI-MS, respectively (Figures S1–S5). In particular, the ^1^H NMR spectra of complex **1** and the DPPI ligand reveals that the TTA^−^ ligand and the rare earth ion [Ln^3+^] are in a stipulated molar ratio of 3:1. In addition, the characteristic proton resonances (*δ* = 13.68 ppm) of the typical group ‘-NH’ relative to the DPPI ligand moved 0.11 ppm to the higher field of complex **1** (*δ* = 13.59 ppm), due to the coordination of the La^3+^ ion by the metal-atomic effects [28,29]. Moreover, the ESI-MS spectra of these typically binary tris-*β*-diketonate-Ln^3+^-complexes show standard molecular ion peaks at 1288.97, 1302.03, 1308.99, and 1307.32, respectively (Ln = La, **1**; Ln = Eu, **2**; Ln = Tb, **3**; or Ln = Gd, **4**) [30,31,32].

**Figure 1:**
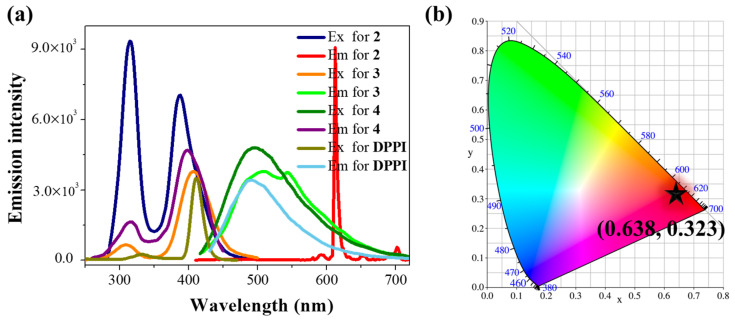
(**a**) Emission and excitation spectra of complexes **2**–**4** and DPPI and (**b**) CIE coordinates of complex **2** in DMSO solution (10^−5^ M) at room temperature (at around *λ*_ex_ = 400 nm, and *λ*_em_ = 612 nm for complex **2**, *λ*_em_ = 545 nm for complex **3**, *λ*_em_ = 495 nm for complex **4** and DPPI).

The photophysical properties of the complexes were examined in dilute DMSO solution at RT or 77 K, and are summarized in Figure 1, Figures S6 and S7 and Table S1. In Figure S6, the similar solution absorption spectra at the ranges of 245–247, 286–288 and 325–380 nm of Ln(TTA)_3_(DPPI) (Ln = Eu, **2**; Ln = Tb, **3**; or Ln = Gd, **4**) complexes are observed, which were contributed to by the ^1^π-π* transitions and the intra-ligand charge transfer (ILCT) bands of the TTA and DPPI ligands, simultaneously [33,34].

For complex **2**, the luminescent emission (Figure 1) shows the Eu^3+^-centered characteristic peaks (^5^D_0_–^7^F*_J_*, *J* = 0–4) with the maximum at 612 nm [26], giving the pure red-light CIE coordinate of (x = 0.638, y = 0.323) and a quantum yield of (*Φ*_Eu_^L^ = 38.9%). For complex **3**, photoexcitation (*λ*_ex_ = 406 nm) gave a weak Tb^3+^-centered characteristic emission at 545 nm, while a strong and wide residual emission (*λ*_em_ = 506 nm) assigned to the DPPI ligand was observed [35]. However, in contrast to complexes **2** and **3** at RT, complex **4** shows that the 0-0 transition phosphorescence (*λ*_em_ = 519 nm, *τ* = 11.3 μs) was under 77 K (Figure S7), assigned to the (^3^π-π*) triplet energy level (20198 cm^−1^) of the ligand. Meanwhile, the (^1^π-π*) singlet energy level (25313 cm^−1^) was obtained by the lowest wavelength of the UV-visible edge. As a result, an energy gap of ΔE (^1^π-π* → ^3^π-π* = 5115 cm^−1^) greater than 5000 cm^−1^ was obtained [36], as shown in Figure S8, which enabled an effective ISC (intersystem crossing) according to Reinhouldt’s empirical rule [37]. Furthermore, through the check of the energy level match between the ligand and the first excited state level of ^5^D_0_ (17286 cm^−1^) of the Eu^3+^ ion, the suitable energy gap ΔE of 2912 cm^−1^ within a 2500–4500 cm^−1^ range from Latva’s empirical rule confirmed the effective sensitization of Eu^3+^. However, the triplet energy level of the ligand was lower than ^5^D_4_ (20545 cm^−1^) of Tb^3+^, and the excessive nonradiative transitions resulted in the quenching of the Tb^3+^-centered effective emission [20]. The Eu^3+^-based (*λ*_em_ = 612 nm) lifetime of **2** was found to be *τ*_obs_ = 217 μs, and a high overall quantum yield (*Φ*_Eu_^L^ = 38.9%) was obtained, further manifesting the efficient energy transfer from the ligand. Noticeably, the Gd-complex **4** also gave an attractive quantum yield (*Φ*_em_ = 8.9%) of blue light emission (x = 0.211 and y = 0.219) from the organic ligands TTA and DPPI. Moreover, the TG analysis of the Ln-complex revealed good thermal stability to be about 300 °C (Figure S9).

### 2.2. Adjustable Color Emission of Complex 2 with TEA Stimulate 

**Figure 2:**
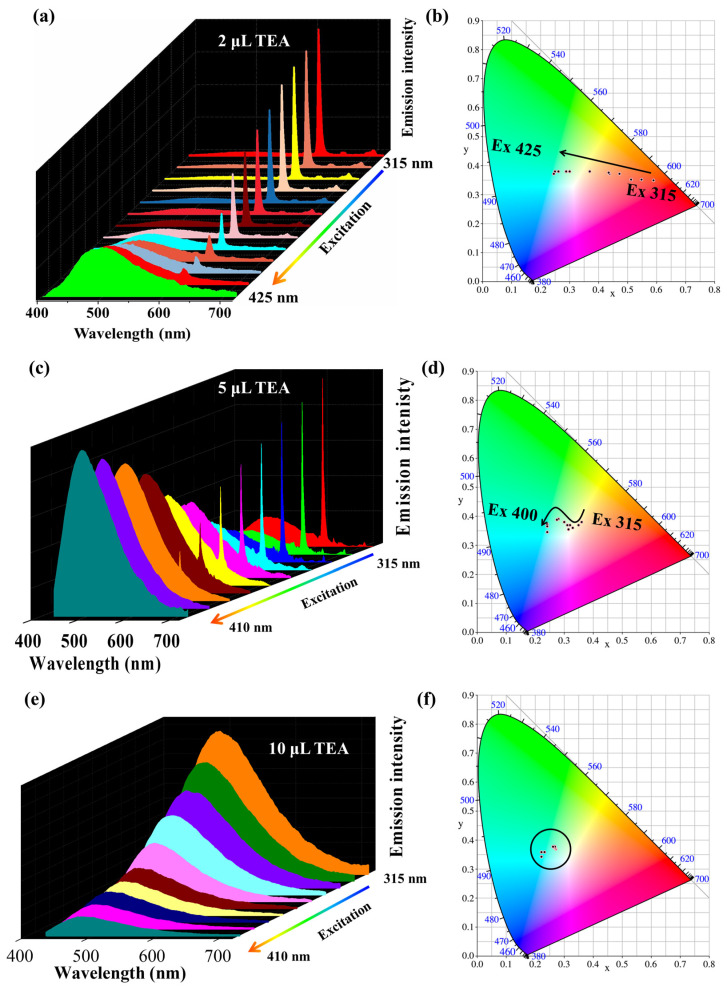
Emission spectra of complex **2** in DMSO solution (1 × 10^−5^ M) stimulated by 2 μL (**a**), 5 μL (**c**) and 10 μL TEA (0.1 mM) (**e**) dependent on the excitations (*λ*_ex_ = 315–425 nm, each interval of 10 nm) at RT. (**b**,**d**,**f**) corresponding CIE graph dependent on the excitations.

As shown in Figure 2a,b, the Eu^3+^-centered red-luminescence intensity of complex **2** in **a** DMSO solution gradually decreased when TEA is added dropwisely. Noticeably, a new broad blue-emission peak (*λ*_em_ = 500 nm) appeared that was dependent on the different excitations and ranged from 315–420 nm. Near white light emission (x = 0.381, y = 0.382) was successfully constructed through the two emission color components (red and bluish green) at the excitation of 385 nm, and the white light emission index parameters are summarized in Table S2. In addition, the fluorescence photographs record the process of the emitting color change upon the addition of different amounts of TEA under ultraviolet light (365 nm), which is shown in Figure S10. As the amounts of TEA increased, the emission intensity of complex **2** at 613 nm gradually weakened and disappeared (Figure 2c–f); meanwhile, the blueish green light emission intensity at around 500 nm gradually increased. When the content of TEA reached 10 μL, it is recorded that the intensity of the residual ligand fluorescence at around 495 nm became the main emission in the whole spectra, and the Eu^3+^-centered emission was very weak and even non-observable.

**Figure 3:**
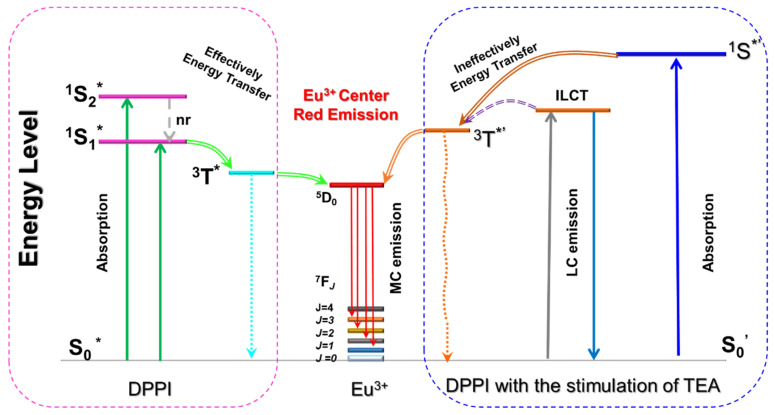
Schematic representation of the energy states and emission processes in the Eu^3+^-complex with the simulation of TEA or not. (* excited state; ’ differentiate the energy states before and after added TEA; LC, ligand-centered; MC, metal-centered; ILCT, intra-ligand charge transfer; S, singlet state; T, triplet state, ET, energy transfer).

To probe the origins of the above emission responses, we speculate that as the TEA ratio in the solution increased, the electronic distribution and intra-ligand charge transfer state between the donor-acceptor groups on the DPPI ligand was changing gradually, accompanying the changes of energy transfer from the DPPI to the Eu^3+^ metal centers [38]. Basically, due to the stimulation of TEA, the energy level of DPPI was elevated (Figures S10–S12, as confirmed by the UV-visible absorption titration tests discussed below), and this caused an incomplete energy transfer to the Eu^3+^ center. Furthermore, the energy transfer processes are sensitive to the excitations. As discussed before, the DPPI ligand absorbs the UV light by either π-π* and/or ILCT singlet states. After transitioning to the triplet states via intersystem crossing (ISC), the energy can be further transferred to the metal centers and can generate the red Eu^3+^-emissions. However, the bluish green emission of the DPPI ligand can only be excited by the ILCT spectral region (see Figure 1a) [39,40]. As shown in the emission spectra of complex **2** at different excitations without TEA (Figure S13), basically only the Eu^3+^-centered emissions were observed, manifesting that the emission of the DPPI ligand itself in complex **2** was hampered due to the total energy transfer to the metal center. After the addition of TEA, both the ligand and the Eu^3+^-centered emissions appeared due to the changes in the ligand electronic states and energy transfer processes to the Eu^3+^ center. However, according to Figure S14, it is clear that the Eu^3+^-centered emission (recorded at 613 nm) can be excited by both the π-π* and ILCT states (showing two excitation peaks), while the ligand-centered emission can only be excited by the ILCT states (basically only one excitation peak is observed). As a result, the alternative intensity between the Eu^3+^-centered and residual fluorescence of the DPPI ligand can be tuned by applying different excitation wavelengths in complex **2** after the addition of TEA, accompanying with various color emissions as a whole (Figure 3).

As shown in Figures S11–S14, in order to further verify the mechanism of the fluorescence changes, the UV-visible absorption titration of **2** upon TEA addition was taken. It can be seen that the absorbance intensity at 230, 270, 285 and 340 nm was obviously enhanced. At the same time, comparing the spectra before and after titration, there was a significant blue shift for the long wavelength absorption peak of nearly 20 nm after the addition of TEA, which shifted from 369 to 349 nm. For comparison, a red-shift tendency was observed upon the addition of acetic acid into the DMSO solution of complex **2**. Such phenomena further confirms that the basic-attributed TEA can cause electronic redistribution in the ligand DPPI, increase its energy levels and regulate the energy transfer from the ligand to the Eu^3+^-metal centers [41].

To further strengthen the reason for the fluorescence alternating processes, dynamic ^1^H NMR spectra were performed for complex **1** (Figure S15). The results recorded that with the addition of TEA, the proton resonances of the typical group ‘-NH’ (*δ* = 13.59 ppm) were gradually widened or weakened. Meanwhile, the whole chemical shift peaks shifted slightly, due to the intermolecular forces between the Ln-complexes and TEA [42,43]. These observations can be attributed to the unique structure of DPPI with imidazole and triphenylamine groups, which are sensitive to the micro-environments changed by the addition of TEA [44,45,46]. On one hand, TEA provides active hydrogen protons that result in acid-base interactions; on the other hand, it causes the electronic distribution and energy state changes in the ILCT-structured DPPI ligand due to molecular interactions [47], and leads to the color emission variations and the TEA detecting capabilities of the Eu^3+^-complex [48,49].

### 2.3. Potential Application in IMPLICATION Logic Gate

**Figure 4:**
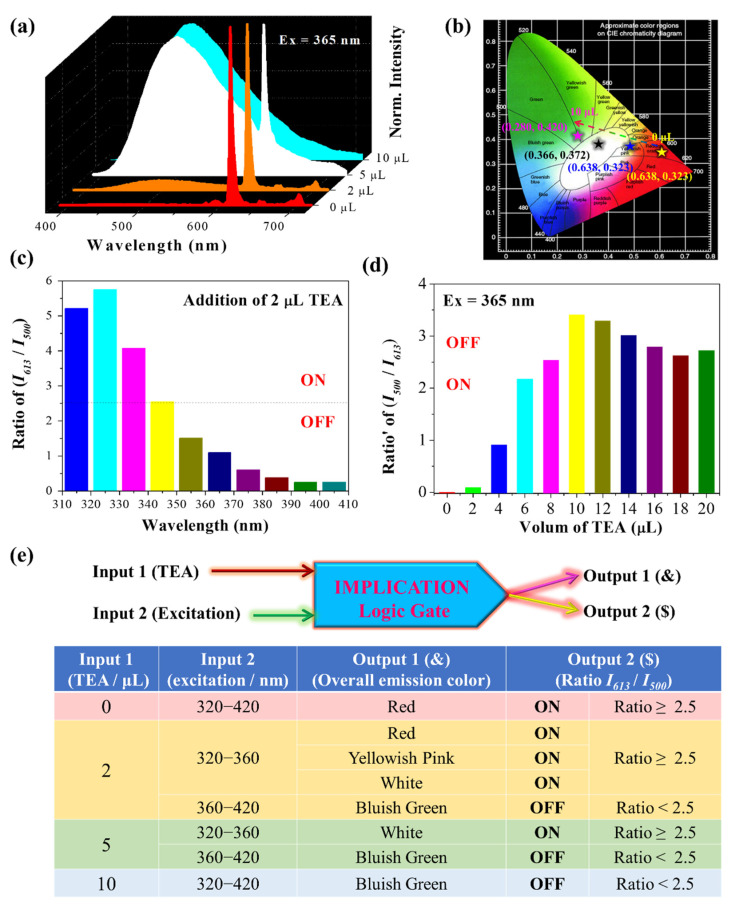
Visible emission spectra (**a**), and corresponding CIE graph (**b**) of complex **2** in DMSO solution stimulated by different amounts of TEA at 1 × 10^−5^ M at RT with the excitation 365 nm. (**c**) corresponding histograms of the fluorescence intensity ratio (R) of **2** at 613 and 500 nm (R = I_613_/I_500_) with 2 μL TEA at different excitation. (**d**) corresponding histograms of the fluorescence intensity ratio (R’) of **2** at 613 and 500 nm (R = I_500_/I_613_) with different amounts of TEA at *λ*_ex_ = 365 nm. (**e**) The IMPLICATION logic gate dependent on the excitation and amounts of TEA as input signals, and the color of the emission and the ratio intensity as dual output signals.

As discussed above, by increasing the amounts of TEA into the solution of complex **2** at the same excitation wavelength (*λ*_ex_ = 365 nm), the color tunable emission can be obtained, which can range from a red emission with a CIE chromatic coordinate of (x = 0.638, y = 0.323), to a white light emission (x = 0.366, y = 0.372), then to a green emission (x = 0.280, y = 0.420) (Figure 3 and Figure 4). The amounts of TEA may affect the rate and degree of the molecular interaction between the TEA molecule and the DPPI ligand, further leading the emission color to change, combined with the results in Figure 2 and Figure 3 and Figures S11 and S12. This is a key factor in determining the fold of the ratio metric enhancement between the two emission peaks at 613 nm and 500 nm of complex **2**. The strongest fluorescence intensity for Eu^3+^ at 613 nm of **2** was achieved without the addition of TEA under *λ*_ex_ = 365 nm. With the addition of TEA, the emission intensity at 613 nm decreased, while the fluorescence intensity at 500 nm assigned to the ligand was gradually enhanced. The corresponding histograms of ratiometric signals vs. various excitations and TEA values are displayed in Figure 4c,d. To this end, the intensity ratio (R = I_613_/I_500_) between the Eu^3+^ emission at 613 nm and the metal disturbed ligand emission at 500 nm is set as the standard, with a threshold of 2.5. If the luminescence ratio value is higher than the default threshold, the ratiometric signals show turn-on. When the ratio is smaller than the threshold, it can be described as the turn-off state.

It is noted that such color tunable changes of fluorescence intensity are dependent both on the excitation wavelength and amounts of TEA, which are conducive to the construction of the logic gate as illustrated in Figure 4e. The following interpretation of two input modes and two output modes can be applied: for input modes, (a) changing the amounts of TEA, and (b) changing the excitation wavelengths. In addition, for output modes: (a) viewing the overall emission color, and (b) detecting the readout ratios for comparing the relative intensities at 613 and 500 nm. As we can see, with different input signals, the output 1 signals (overall emission color) can be red, yellowish pink, white, or blueish green, while the output 2 signals will change from ON (I_613_/I_500_ > 2.5) to OFF (I_613_/I_500_ < 2.5). Therefore, we can design a dual-input/dual-output color IMPLICATION logic gate.

## 3. Experimental Section

### 3.1. Synthesis of 1,10-Phenanthroline-5,6-Dione (PHD)

The general procedure for the synthesis of PHD followed the methods in the literature [50]. An ice-cooled mixture solution of 20 mL H_2_SO_4_ and 10 mL HNO_3_ was added by (11.0 mmol, 2.0 g) 1,10-phenanthroline and (16.8 mmol, 2.0 g) KBr, stirred at room temperature for 20 min. After this procedure, the mixture was stirred at 130 °C for another 3 h. Then the hot yellow solution was poured into 500 mL of ice water and neutralized by a saturated Na_2_CO_3_ aqueous solution until pH = 7–8. The solution was then extracted with CHCl_3_, dried with anhydrous Mg_2_SO_4_ and the solvent was removed. There was a yield of 98%. ^1^H NMR (400 MHz, DMSO-*d*_6_): δ 9.05 (d, 2H, -pyridyl), 8.56 (d, 2H, -pyridyl), 7.84 (t, 2H, -pyridyl). FT-IR (KBr, cm^−1^): 1684 (s), 1560 (m), 1459 (w), 1414 (s), 1313 (w), 1293 (s), 1205 (w), 1115 (w), 1010 (w), 925 (w), 807 (m), 736 (vs), 668 (w), 613 (w), 540 (w). The elemental analysis calculated (%) for C_12_H_6_N_2_O_2_ was C, 68.57; H, 2.88; N, 13.33. C, 68.49; H, 2.77; N, 13.21. MS (ESI^+^): *m*/*z*: 211.04(100%), [M − H]^+^ was found. 

### 3.2. Synthesis of DPPI

The DPPI was synthesized by refluxing a mixture of 1,10-phenanthroline-5,6-dione (0,21 g, 1 mmol), 4-(diphenylamino)-benzaldehyde (0.27 g, 1 mmol), NaH_2_PO_4_ (0.012 g, 0.1 mmol) and ammonium acetate (1.54 g, 20 mmol) in glacial acid (40 mL) for 6 h under a N_2_ atmosphere, according to the literature [51]. After being stirred overnight, the mixture was cooled to room temperature, and then the aiming compound was obtained by the addition of a K_2_CO_3_ solution to the mixture filtration and from washing with enough water. The crude product was further extracted by CHCl_3_ and vacuum dried. There was a yield of 46%. ^1^H NMR (400 MHz, DMSO-*d*_6_): δ 13.68 (s, 1H, -NH), 9.04 (d, 2H, -pyridyl), 8.91 (d, 2H, -pyridyl), 8.17 (t, 2H, -pyridyl), 7.83 (m, 2H, -phenyl), 7.45–7.36 (m, 4H, -phenyl), 7.25–7.07 (m, 8H, -phenyl). FT-IR (KBr, cm^−1^): 3060 (w), 1590(m), 1521 (w), 1480 (s), 1450 (w), 1397 (w), 1317 (w), 1280 (m), 1190 (w), 1126 (w), 1069 (w), 1029 (w), 952 (w), 840 (w), 804 (w), 740 (s), 694 (vs), 636 (w), 619 (w), 530 (w). The elemental analysis calculated (%) for C_25_H_23_N_3_ was C, 85.87; H, 5.02; N, 9.10. C, 86.06; H, 5.18; N, 9.37. MS (ESI^+^): *m*/*z*: 486.17(100%), [M − H]^+^ was found. 

### 3.3. Synthesis and Characterization of Ln(TTA)_3_(DPPI) (Ln = La, 1; Ln = Eu, 2; Ln = Tb, 3; Ln = Gd, 4)

A solution of 2-Thenoyltrifuoroacetone (HTTA) (0.20 g, 0.9 mmol), and sodium hydroxide (0.36 g, 0.9 mmol) was added in 5 mL of mixed solvent (MeOH/chloroform = 3: 5) and stirred for 30 min. Then DPPI (0.13 g, 0.3 mmol) was added, and then heated at 50 °C for 30 min with stirring. Following this, the solution of LnCl_3_-6H_2_O (0.3 mmol; Ln = La, 0.10 g; Ln = Eu, 0.11 g; Ln = Tb, 0.12 g or Ln = Gd, 0.11 g) was dissolved in 2 mL of MeOH and added dropwise and stirred at 50 °C for 12 h. The light yellow product was isolated by precipitation in ice-cold *n*-hexane and dried for 24 h in a vacuum oven at 30 °C to obtain a milk-white powder. The detailed process is shown in Scheme 1. The series of complexes Ln(TTA)_3_(DPPI) (Ln = La, **1**; Ln = Eu, **2**; Ln = Tb, **3**; Ln = Gd, **4**) were obtained and well-characterized by EA, FT-IR, ^1^H NMR and ESI-MS, respectively.

For **1** (C_55_H_33_F_9_O_6_N_5_S_3_La), ^1^H NMR (400 MHz, DMSO-*d*_6_): δ 13.59 (s, 1H, -NH), 9.05–8.91 (t, 4H, -phenyl, and -pyridyl), 8.17 (d, 2H, -phenyl), 7.83 (m, 8H, -phenyl, -thienyl and -pyridyl), 7.38 (m, 4H, -phenyl, -thienyl and -pyridyl), 7.15–7.12 (m, 11H, -phenyl, -thienyl and -pyridyl), 6.17 (s, 3H, -CH=C-). FT-IR (KBr, cm^−1^): 1599 (s), 1536 (s), 1481(m), 1454(m), 1411 (s),1355 (w),1298 (vs), 1246 (w),1229 (w), 1186 (s), 1137 (s), 1078 (w), 1061 (w), 933 (w), 859 (w), 842 (w), 786 (m), 767 (w), 718 (w), 696 (m), 680 (w), 640 (m), 604 (w), 579 (m), 520 (w). The elemental analysis (%) calculated for C_55_H_33_F_9_O_6_N_5_S_3_La was C, 52.18; H, 2.63; N, 5.53. C, 52.21; H, 2.49; N, 5.60. MS (ESI^+^): *m*/*z*: 1288.97(100%), [M + Na]^+^ was found. The yield was 60%.

For **2** (C_55_H_33_F_9_O_6_N_5_S_3_Eu), FT-IR (KBr, cm^−1^): 1594 (s), 1537 (s), 1505 (m), 1454 (m), 1410 (s),1354 (w),1306 (vs), 1247 (w),1229 (w), 1181(s), 1135 (s), 1076 (w), 1062 (w), 933 (w), 860 (w), 842 (w), 788 (m), 768 (w), 719 (w), 696 (m), 683 (w), 640 (m), 605 (w), 579 (m), 521 (w). The elemental analysis (%) calculated for C_55_H_33_F_9_O_6_N_5_S_3_Eu was C, 51.65; H, 2.60; N, 5.48. C, 51.62; H, 2.59; N, 5.46. MS (ESI^+^): *m*/*z*: 1302.05(100%), [M + Na]^+^ was found. The yield was 63%.

For **3** (C_55_H_33_F_9_O_6_N_5_S_3_Tb), FT-IR (KBr, cm^−1^): 1594 (s), 1537 (s), 1505 (m), 1454 (m), 1410 (s),1354 (w),1306 (vs), 1247 (w),1229 (w), 1181(s), 1135 (s), 1076 (w), 1062 (w), 933 (w), 860 (w), 842 (w), 788 (m), 768 (w), 719 (w), 696 (m), 683 (w), 640 (m), 605 (w), 579 (m), 521 (w). The elemental analysis (%) calculated for C_55_H_33_F_9_O_6_N_5_S_3_Tb was C, 51.37; H, 2.59; N, 5.45. C, 51.29; H, 2.52; N, 5.39. MS (ESI^+^): *m*/*z*: 1308.99(100%), [M + Na]^+^ was found. The yield was 65%.

For **4** (C_55_H_33_F_9_O_6_N_5_S_3_Gd), FT-IR (KBr, cm^−1^): 1598 (s), 1538 (s), 1480 (m), 1454 (m), 1411 (s),1355 (w), 1304 (vs), 1247 (w),1230 (w), 1188 (s), 1137 (s), 1078 (w), 1062 (w), 934 (w), 860 (w), 842 (w), 787 (m), 768 (w), 718 (w), 695 (m), 681 (w), 641 (m), 605 (w), 581 (m), 521 (w). The elemental analysis (%) calculated for C_55_H_33_F_9_O_6_N_5_S_3_Gd was C, 51.44; H, 2.59; N, 5.45. C, 51.42; H, 2.53; N, 5.39. MS (ESI^+^): *m*/*z*: 1307.32 (100%), [M + Na]^+^ was found. The yield was 61%.

## 4. Conclusions

In summary, a purity-red emitter (x = 0.638, y = 0.323; *Φ*_Eu_^L^ = 38.9%) of Eu^3+^-complex was synthesized, which showed color tunable emissions that ranged from red, white, to bluish green under the different excitation wavelengths and various amounts of TEA stimulations. Relying on the changeable color emission, we successfully designed the dual-input/dual-output logic gate for the visual detection of toxic TEA in a solution, which expands the research applications of lanthanide Eu^3+^ luminescent materials.

## Data Availability

All data are included in the article.

## Supplementary Materials

The following are available online. Figure S1 Partial ^1^H NMR spectra of PHD, DPPI and complex 1. Figure S2 FT-IR spectrum of DPPI. Figure S3 FT-IR spectra of complexes 1–4. Figure S4 ESI mass spectrum of DPPI. Figure S5 ESI mass spectrum of complex 2. Figure S6 UV-visible absorption spectra of complexes 2–4, the ligand HTTA and DPPI. Figure S7 Emission and excitation spectra of complex 4 at 77 K. Figure S8 Energy transfer process from DPPI ligand to Eu^3+^ or Tb^3+^ in complexes 2–3. Figure S9 TG curves of 2 and DPPI. Figure S10 Fluorescent photographs of complex 2 under Sunlight and UV light. Figure S11 UV-visible absorption spectra changes of complex 2. Figure S12 UV-visible absorption spectra changes of complex 2 with the stimulation of acetic acid (0.1 mM) in DMSO solution (10^−5^ M) at RT. Figure S13 Emission spectra of complex 2 by different excitation wavelengths (λ_ex_ = 290 to 410 nm) in DMSO solution without TEA. Figure S14 Excitation spectra of complex 2 monitored at 495 nm and 613 nm with the simulation of TEA in solution at room temperature. Figure S15 ^1^H NMR (400 MHz, DMSO-*d*_6_) of complex 1 with the addition of TEA (0.1 mM). Table S1 Photophysical properties of complex monomers 2–4, HTTA and DPPI. Table S2 White light emission index parameters.

## Sample Availability

Samples of the compounds DPPI and complex **2** are available from the authors.
