# Supplementary material for: Visual Detection of Triethylamine and a Dual Input/Output Logic Gate Based on a Eu3+-Complex
Source: Molecules. 2021 May 28;26(11):3244. doi: 10.3390/molecules26113244 (PMC8198769; doi:10.3390/molecules26113244)
Supplement: Supplementary file 1 [file molecules-26-03244-s001.zip › molecules-1215217-supplementary.pdf]

molecules

MDPI

Supplementary Materials

Visual Detection of Triethylamine and a Dual Input/Output Logic Gate Based on a Eu^{3+}–Complex

Bao-Ning Li, Yuan-Yuan Liu, Ya-Ping Wang and Mei Pan *

Materials and methods

High performance liquid chromatography (HPLC)−grade DMSO was purchased from Fisher Scientific and purified over solvent columns. Other solvents were used as received from Sigma Aldrich and stored over 3 Å activated molecule sieves. Other chemicals were commercial products of reagent grade, and were used without further purification. All manipulations of air and water sensitive compounds were carried out under dry N_2 using the standard Schlenk line techniques. ^1H NMR spectra were recorded on a Bruker DPX 400 FT–NMR spectrometer 400 MHz at 298 K. Electrospray ionization mass spectra (ESI−MS) were performed on a Bruker Maxis 4G ESI–Q–TOF Bruker using electrospray ionization (positive ion mode: ESI) in solution. Fourier Transform Infrared (FT−IR) spectra were recorded on a Nicolet Nagna−IR 550 spectrophotometer in the region 4000−400 cm^{-1} using KBr pellets. Elemental analyses of the complexes were made using a PerkinElmer 240 elemental analyzer. Electronic absorption spectra in the UV−visible region were recorded with a Shimadzu UV−3600 spectrophotometer, in a 10×1 mm or 10×10 mm quartz cell. Visible emission and excitation spectra were collected by a combined fluorescence lifetime and steady-state

spectrometer (FLS−980, Edinburgh) with a 450 W Xe lamp. Excited-state decay times were obtained by the same spectrometer but with a *μ*F 900 Xe lamp. The luminescent absolute overall quantum yield (Φ_{em}^{L}) was determined by the same spectrometer using a 450 W *X*e lamp and an integrating sphere. Thermal properties were characterized using Thermogravimetric (TG) analyses on a NETZSCH TG 209 instrument under flowing nitrogen at a heating rate of 10 °C/min.

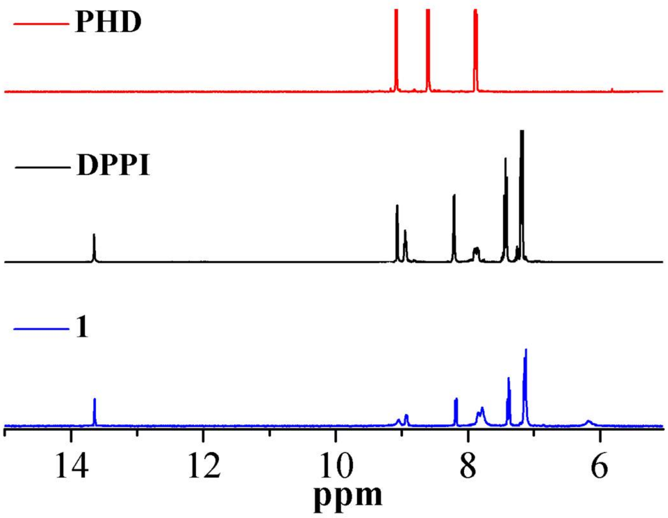

Figure S1 Partial ^{1}H NMR spectra of PHD, DPPI and complex **1** in DMSO−d_6 at room temperature.

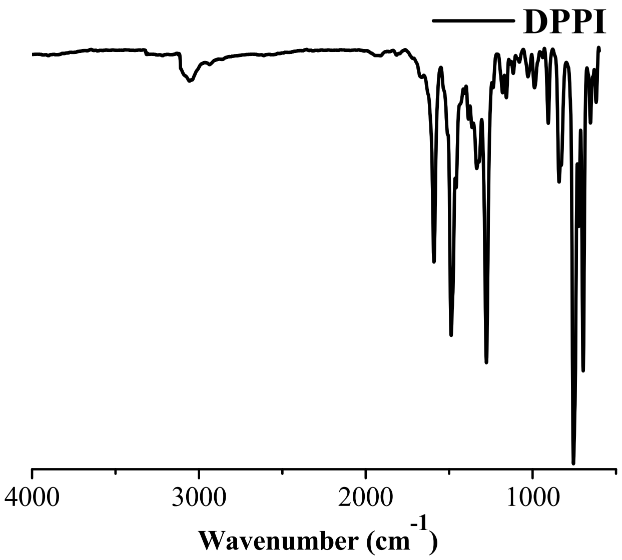

Figure S2 FT−IR spectrum of DPPI in solid state at room temperature.

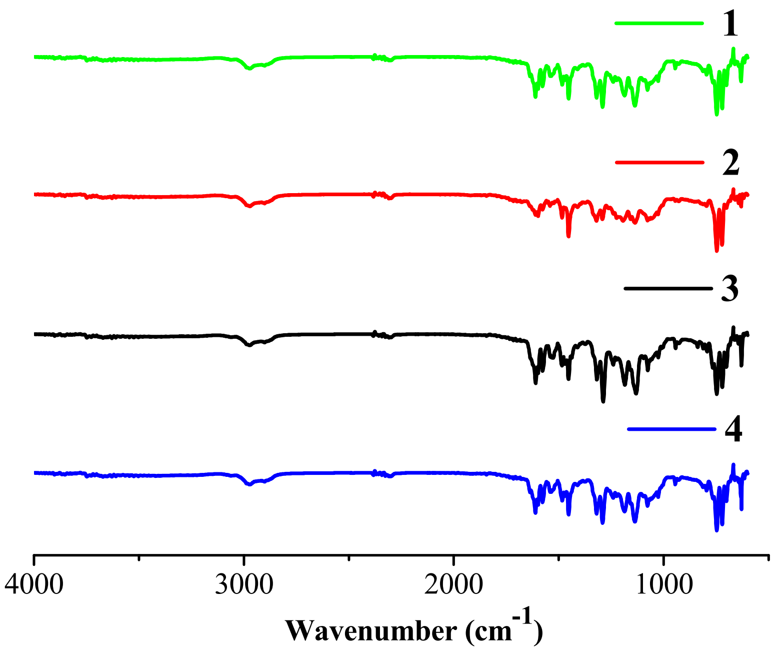

Figure S3 FT−IR spectra of complexes **1−4** in solid state at room temperature.

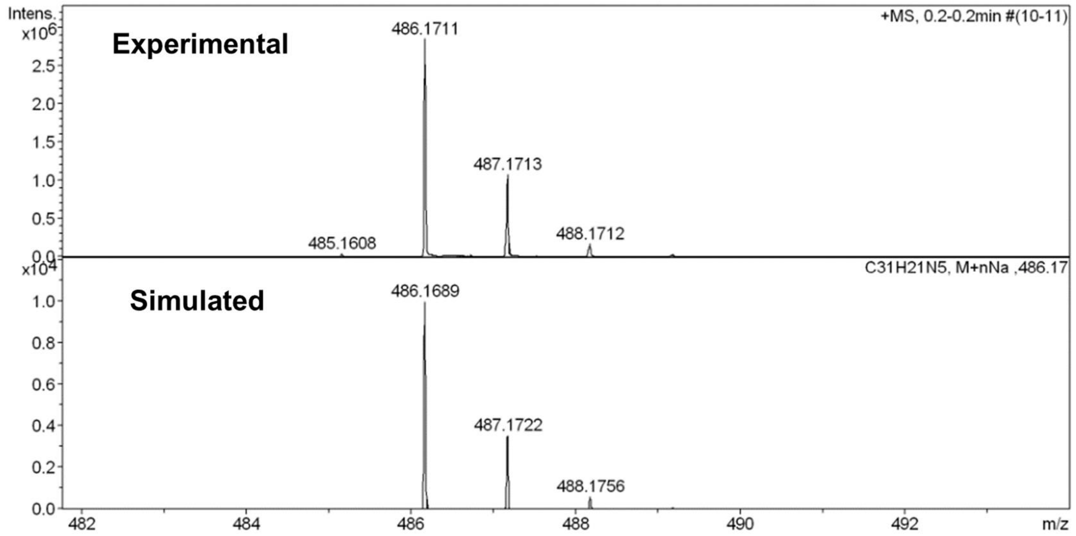

Figure S4 ESI mass spectrum of **DPPI** in MeCN solution at room temperature.

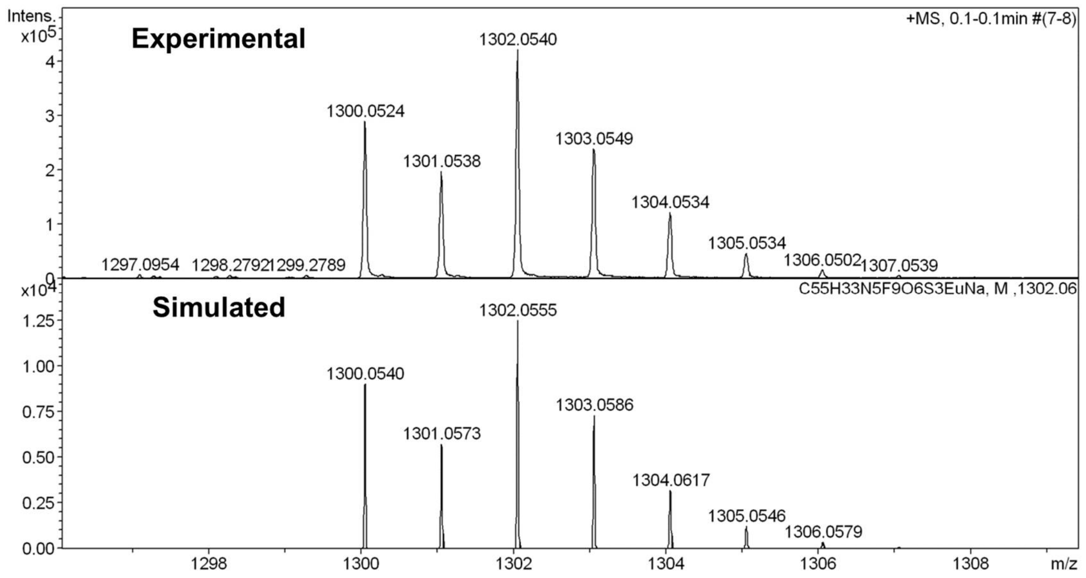

Figure S5 ESI mass spectrum of complex **2** in MeCN solution at room temperature.

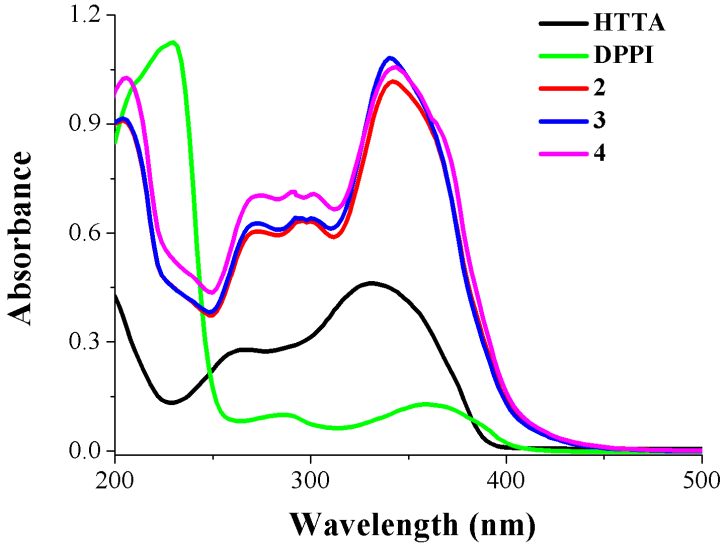

Figure S6 UV-visible absorption spectra of complexes **2**−**4**, the ligand HTTA and DPPI in dilute DMSO solution (1×10^{-5} M) at room temperature.

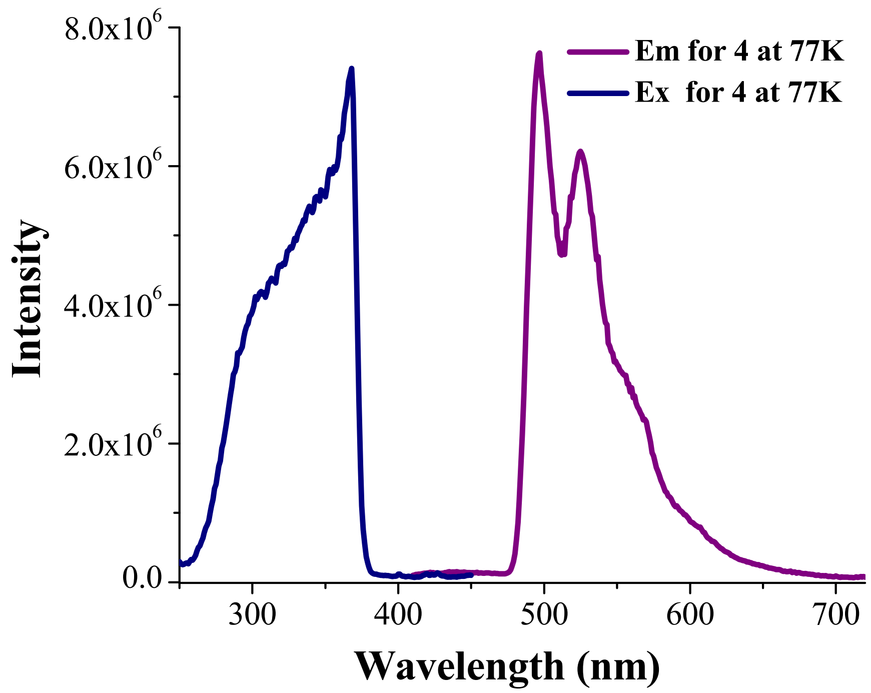

Figure S7 Emission and excitation spectra of complex **4** in DMSO solution (1×10^{-5} M) at 77 K.

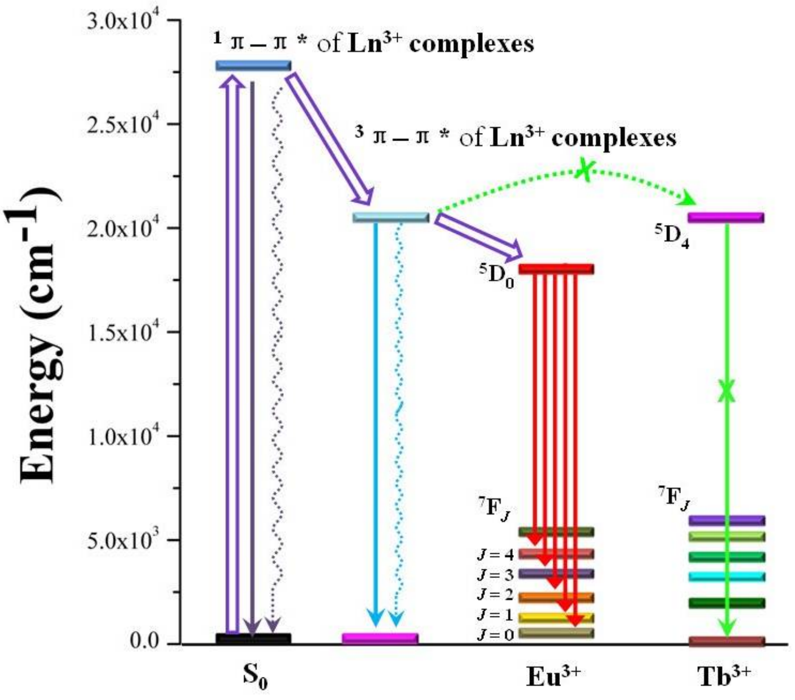

Figure S8 Energy transfer process from DPPI ligand to Eu^{3+} or Tb^{3+} in complexes **2**–**3**.

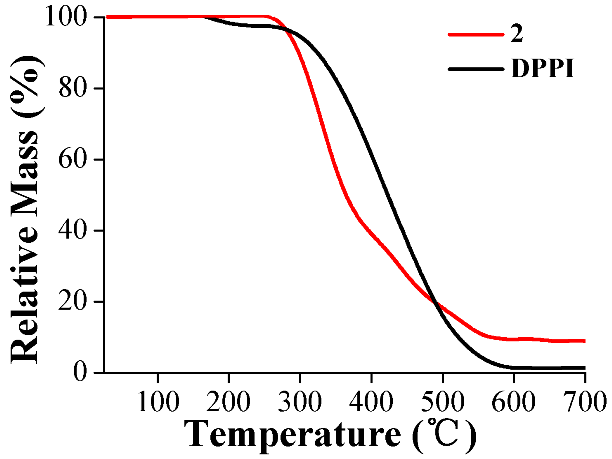

Figure S9 TG curves of **2** and DPPI in solid state under N_2 atmosphere.

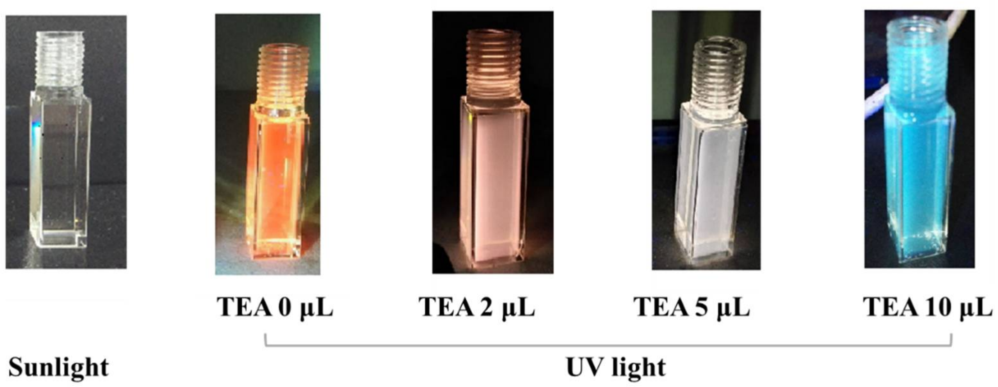

Figure S10 Fluorescent photographs with the different amounts of TEA (0.1 mM) stimulation of complex **2** (10^{-5} M) under Sunlight and UV light.

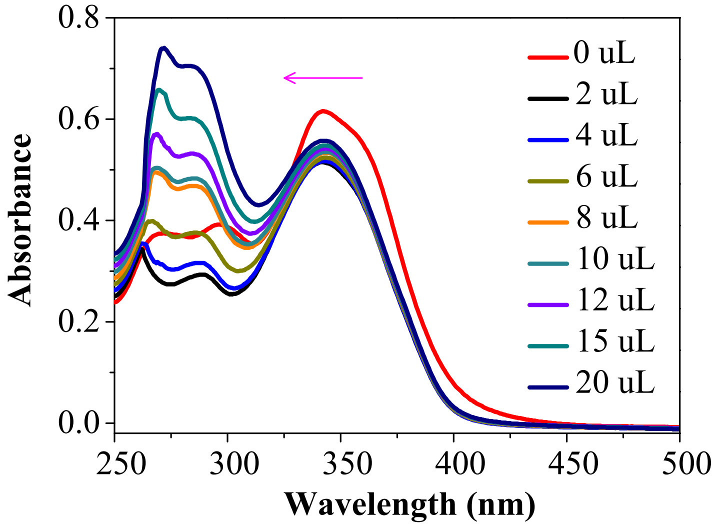

Figure S11 UV−visible absorption spectra changes of complex **2** with the stimulation of TEA (0.1 mM) in DMSO solution (10^{-5} M) at RT.

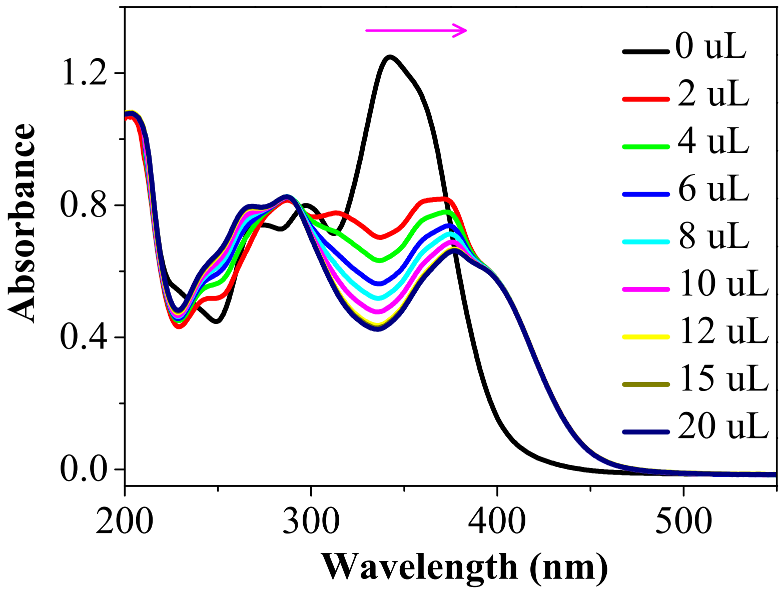

Figure S12 UV−visible absorption spectra changes of complex **2** with the stimulation of acetic acid (0.1 mM) in DMSO solution (10^{-5} M) at RT.

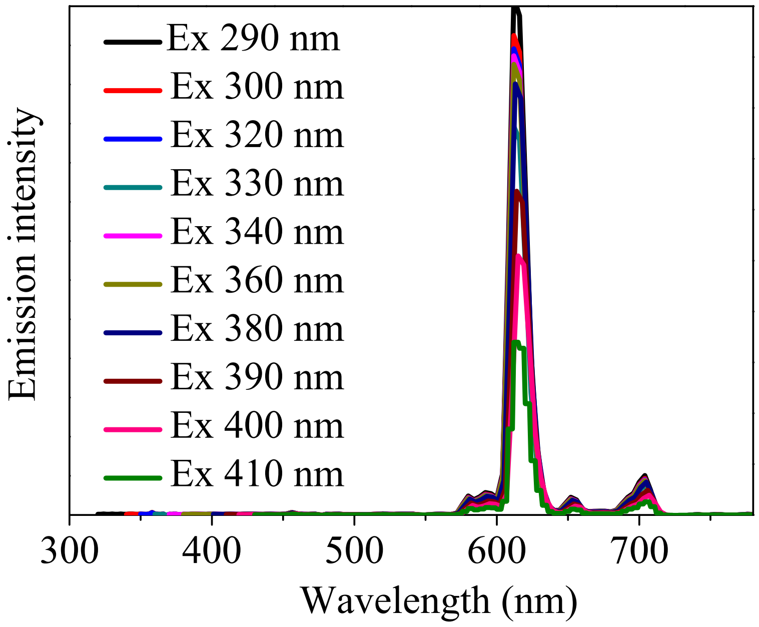

Figure S13 Emission spectra of complex **2** by different excitation wavelengths (λ_{ex} = 290 to 410 nm) in DMSO solution without TEA.

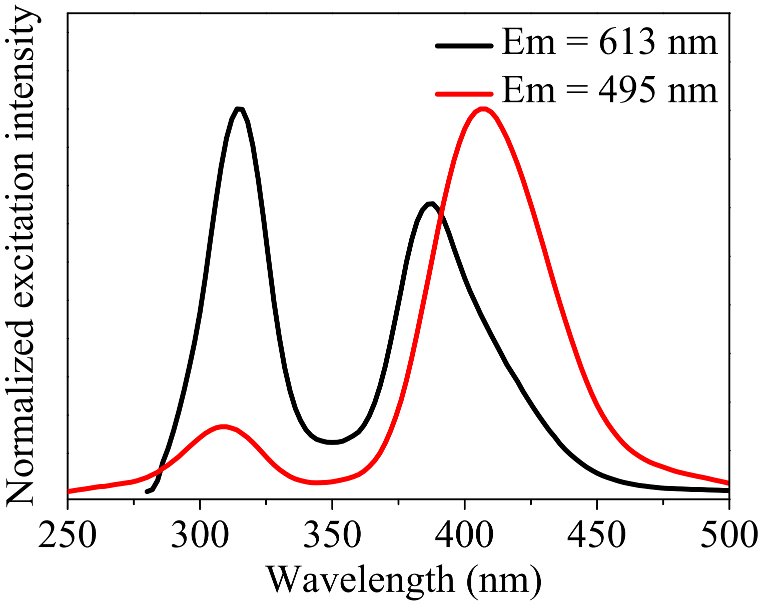

Figure S14 Excitation spectra of complex **2** monitored at 495 nm (red curve) and 613 nm (black curve) with the simulation of TEA in solution at room temperature.

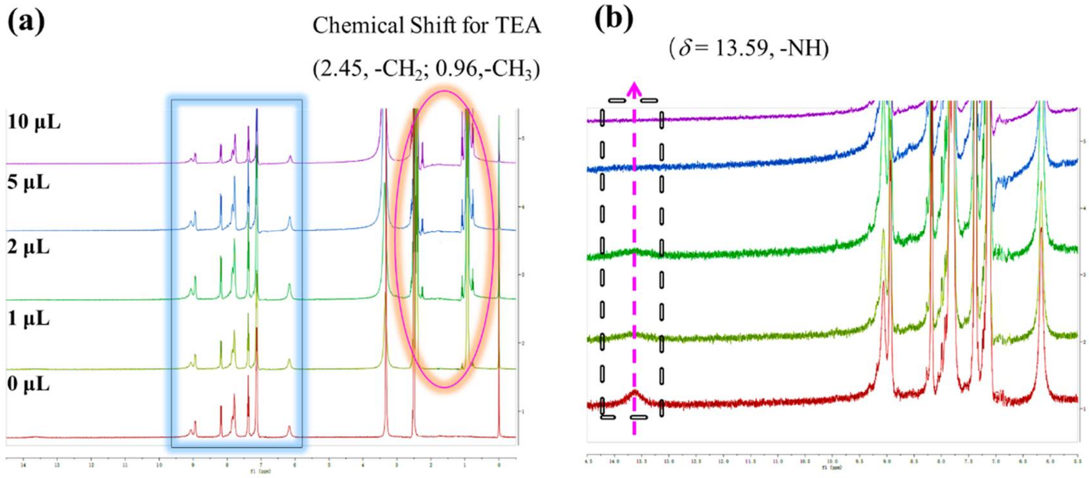

Figure S15 (a) ^{1}H NMR (400 MHz, DMSO−d_6) of complex **1** with the addition of TEA (0.1 mM). (b) Enlarged image from 14.5 to 5.5 ppm in order to observe the active hydrogen obviously).

Table S1 Photophysical properties of complex monomers **2−4,** HTTA and DPPI in dilute DMSO solution (1×10^{-5} M) at RT or 77 K.

**Compounds**	λ_{ab}/nm[log(ε/dm^{3}mol^{-1}cm^{-1})]	λ_{ex}/nm	λ_{em}/nm (τ, Φ (%))
**2**	260(0.69), 290(0.71), 341(0.89)	375	594, 613 (327 μs, 38.9%), 652, 701
**3**	262(0.65), 289(0.76), 342(0.91)	375	545 (2.6 ns), 506 (2.5 ns)
**4**	261(0.68), 291(0.73), 341(0.88)	375	502 (2.0 ns)
**HTTA**	262(1.02), 288(1.12), 333(0.96)	345	519 (11.3 μs)
**DPPI**	229(1.32), 288(0.26), 369(0.37)	375	490

Table S2 White light emission index parameters stimulated by 2 μL TEA under different excitation wavelengths in solution at RT**.**

Complex 2	Ex / nm	CIE	CRI	CCT
2 μL TEA	315	(0.521, 0.340)	11823	10.88
	325	(0.553, 0.344)	11336	11.12
	335	(0.553, 0.345)	10157	10.91
	345	(0.525, 0.350)	7804	9.26
	355	(0.481, 0.361)	5427	12.37
	365	(0.441, 0.362)	3863	16.56
	375	(0.433, 0.371)	2185	21.16
	385	(0.381, 0.382)	3763	38.17
	395	(0.290, 0.382)	5024	58.09
	405	(0.301, 0.383)	7516	73.86
	415	(0.260, 0.380)	7654	65.83
	425	(0.259, 0.383)	7523	60.00

#: In the titration experiment, 2−10 μL TEA adding into 5 mL sample solution, therefore we can ignore the influence of the change of solvent volume.
